# Road to better health and integration: a Delphi study on health service models for Hong Kong migrants

**DOI:** 10.1186/s12939-014-0127-x

**Published:** 2014-12-19

**Authors:** William CW Wong, Petula SY Ho, Jun Liang, Eleanor A Holroyd, Cindy LK Lam, Agnes MY Pau

**Affiliations:** Department of Family Medicine and Primary Care, The University of Hong Kong, 3rd Fl., Ap Lei Chau Clinic, 161 Main Street, Ap Lei Chau, Hong Kong; Department of Social Work and Administration, Faculty of Social Science, The University of Hong Kong, Hong Kong, Hong Kong; Department of Family Medicine, Tuen Mun Hospital and Family Medicine Cluster Coordinator, Hospital Authority New Territories West Cluster, Hong Kong, Hong Kong; Asian and Gender Studies, RMIT University, PO Box 71, Bundoora, VIC 3083 Australia

**Keywords:** Healthcare delivery, Access to Health Care, Health Policy, Migrants, Delphi technique

## Abstract

**Introduction:**

In Hong Kong, migrants arriving from Mainland China often have multiple roles and responsibilities while adapting to new lives in their host destination. This paper explored the factors that contribute to the inequity in health services utilisation experienced by these migrants; and, identified the elements that could constitute an effective health delivery model to address the service gap.

**Methods:**

Site visits and a focus group discussion (n = 13) were held with both public and private health providers before a number of innovative health delivery models were formulated. They were then circulated among the panel in two further rounds of Delphi survey (n = 11) from March-April 2012 to systematically collect opinions and select the most endorsed health service models to serve this target population.

**Results:**

Focus group members perceived that most migrants were unaware of, or even ignored, their own physical and mental health needs, and had low utilisation of healthcare services, because of their pre-occupation with daily chores and hardship as well as differing health values, practices and expectations. They further identified that the structural issues such as the healthcare setting or the operation of current service provisions had failed to meet migrants’ health needs. Consequently, four new service models that incorporated professional advice and empowerment, which were identified as the two most important elements, were put forward. Thus, the model of having a nurse with social work training, supported by volunteer groups, was selected as the best option to familiarise and empower patients within the labyrinth of local healthcare services.

**Conclusion:**

Implementation of a social empowerment model by way of targeted support and specific health information is recommended. Further evaluation of this model is needed to understand its effectiveness for improving health literacy and health status in this disadvantaged group in the long term.

## Introduction

Health inequity refers to disparities in health that are deemed to be unfair stemming from specific forms of injustice which are avoidable or unnecessary [1]. Migration is a well-known social determinant in the health inequity framework. Considerable literature has shown how migration can negatively affect the health and health access of migrants [2,3]. This population is known to be exposed to greater risks of poor physical and mental health because of the changes in their socio-economic status and reduction in utilisation of formal healthcare services [2,4-6]. Although there exists considerable information on the contribution of gender and socio-economic status to the general health status and health service utilisation of the migrants in Hong Kong [7,8], an understanding of how government and policymakers should address the planning and execution of effective of healthcare and social services to meet the specific needs of the migrant populations is limited.

Hong Kong has always been a popular destination for migrants and refugees. In 2011 alone over 43,300 migrants, mainly comprised of women and children, entered Hong Kong through The One-way Permit for family reunification system and, in 2000–2011, a total of 567,319 Mainland Chinese, amounting to 8% of the entire population, came to reside in Hong Kong [9]. In Hong Kong as well as in other parts of Asia and internationally, many citizens voluntarily migrate elsewhere due to push-pull factors such as economic hardship, study opportunities, marriage prospects and family re-unification. Migration for marriage purposes constitutes a large percentage of the reasons for migration among women in China, Southeast and Northeast Asia. These migrating brides often face considerable institutional hurdles and problems of integration in their host societies and are confronted with long and uncertain pathways to citizenship and welfare entitlements [10,11]. These challenges and uncertainties have been attributed to the policymakers’ tendency to view migration as an important source of economic capital in recognition of the migrants’ potential contributions to their host societies capital growth and ability to provide formal and informal health services for the aging populations in the form of family caregivers, skilled and semi-skilled health professionals [12]. Yet these assumed contributions of migrant women are hinged upon their successful integration into their new host society [13].

A recent local study showed that migrants live in lower socio-economic conditions; have poorer health status; and, utilise healthcare services to a lesser degree than other local populations in their host society [14]. Younger migrant women tended to have fewer doctor visits and relied more heavily on the public health sector. Other studies have shown that spousal migrants were more susceptible to acculturative stress resulting from discrimination and that the stress from their “dual” roles as family caregivers and breadwinners might predispose them to higher psychological distress [15,16]. At the same time, mistrust and disappointment arising from unfamiliarity with local healthcare professionals and the host country’s formal health care system were commonly reported and likely contributed to under-utilisation of healthcare services.

The aim of this study was to identify gaps in current health service models and develop new ways that would reduce health inequity experienced by Chinese migrants, especially female migrants, to actualise the full potential of available health services. This paper describes the use of a qualitative approach and a Delphi surveying technique to identify the characteristics of various health service models that would address service gaps and facilitate migrants’ access to healthcare.

## Methods

In order to understand the range and types of services provided for migrant communities, participatory observations whereby the two of the investigators (WW and AMYP) would spend some time sitting in the waiting areas observing their interactions with the clinical staff and other patients and in-depth interviews were conducted during a number of migrant population health service visits, in both the private and public health settings, between 2nd March and 24th April, 2012. First, a private medical centre was targeted for site visits and interviews were conducted with the family physician and allied staff at the location. The medical centre provided general medical care and specialist services such as Ophthalmology, Obstetrics and Gynaecology. Second, the General Outpatient Clinic and Accident & Emergency Department at North District Hospital were visited and follow-up interviews conducted with one doctor-in-charge, one department operation manager, one nursing officer and a selected Patient Care Assistant (PCA) in each location. In addition, the research team conducted observations of the clinical set-up and how migrants interacted with the service providers in these locations.

Following the site visits, a focus group discussion (FDG) was arranged with staff through convenience sampling from each centre from each centre to obtain first-hand views on the health of Chinese migrants, perceived barriers to accessing formal health services and suggestions on how current services might be improved. Participants in the FGD were either in contact with the migrants or had experience with the subject matter (Table 1). A focus group was conducted using a semi-structured interview guide developed from observations and previous interviews and lasted approximately one to one-and-a-half hours. Issues of quality, rigor and trustworthiness in conducting the focus group were addressed based on a framework by Meyrick [17]. The focus group was audio-recorded, and subsequently transcribed.

**Table 1:** **Composition of FGD and Delphi round stakeholders and experts**

	**Focus group participants**	**Delphi survey participants**	**Credentials of participants**
**Social and health policy experts**	1	3	Policy expert; (Ex-)Director of a school of public health of a tertiary institution; Board member of Hospital Authority
**Frontline health professionals**	3	3	Consultant and Associate Consultant of Hospital Authority; Doctor-in-charge of a local NGO clinic
**Social workers**	2	2	Senior Social Workers working with migrants; Social worker (research)
**Clinical Psychologists**	2	-	Working in the newly developed community
**Migrant representative**	1	1	Recruited by a social worker
**Patient representative**	1	1	Deputy Director of patient self-help group
**Teacher**	1	1	Working in an area populated with migrants
**Health Researchers**	2	-	Specialised in health service research
**Total**	13	11	

The transcripts were reviewed and verified by two investigative team members using constant comparative methods [18]. The data were coded independently and analysed when concerns about health status and/or associated problems faced by migrants in gaining health access were identified. Moreover, the service models were generated from relevant literature as well as being grounded in the thematic analysis of FGD data. These models were compared and contrasted by the research team members, and the combined results then informed the Delphi questionnaire. The Delphi questionnaire was subsequently distributed to the expert panel, in order to address the identified problems.

### Delphi technique

The Delphi technique is an organised and structured social marketing method for correlating views and information pertaining to a specific policy area enabling respondents to represent their views and knowledge while having the opportunity to react to and assess differing viewpoints [19,20]. This technique is regarded as a powerful and cost-effective way to obtain information and opinions from a dispersed group of people with various expertises. Typically the Delphi technique requires three rounds of questionnaires to be sent to an expert panel. The role of the panel is to first identify key issues to be addressed in later rounds, while subsequent rounds require more specificity, with the questionnaires seeking quantification of earlier findings [19]. In the reported study, the initial FGD served as the role of the first round wherein key issues were identified. Subsequently a questionnaire with several proposed service models was designed from the collated observations, responses from the site visits and FGD. These two rounds of questionnaires were then sent by e-mail or post to the expert panel made up of 11 members (from the original 13-member FGD panel), each of whom had at least two years of experience working directly on migrant health issues. These “experts” were selected based on their association as key stakeholders with knowledge and insight on migrant health and included social and health policy experts, a researcher working on migrant children, a board member of the Hong Kong Hospital Authority, frontline doctors working at both public and private sectors, social workers that worked with migrants, a patient group representative who happened to be a migrant himself as well as a teacher who taught students at a migrant-populated district in Hong Kong. In order to encourage doctors to participate in the expert panel, Continuing Medical Education points were awarded to those who had taken part in the FGD.

The questionnaire contained several proposed health service models that solicited the expert participants’ opinion on the advantages and difficulties in implementation of each of these proposed models as well as suggested improvements. The participants were asked to identify the qualities or factors of each service model that would contribute to improving migrants’ health access and health status. The questionnaire was further translated into Chinese to accommodate to the needs of the migrant representatives on the panel. A total of 10 (90.9%) questionnaires were received in the first round and the experts’ choices of service models with explanations were collated, with common and conflicting viewpoints identified. The proposed service models were then modified according to the responses collected and further analysed. A total of 7 (63.6%) questionnaires were returned in the second round of surveying.

## Results

### Findings of the visits, interviews and focus group discussion

The major themes identified that contributed to problems faced by migrants when accessing Hong Kong’s formal healthcare facilities included: (1) health literacy and communication; (2) mental health and social support; and (3) accessibility and appropriateness of health service and information (Table 2). Four models of care to address these issues were proposed.

**Table 2:** **Problems and service models generated from FGD and site visits**

**Problems**	**1) Issues on health literacy and different cultural expectations**	**2) Strong need for mental health service and family/social support**	**3) Accessibility and appropriateness of health services and information**
	i. Lack of Primary and Secondary care concepts and result in doctor shopping and non-discriminatory self-medication;	i. Lack of social support from community or family	i. High time and financial cost;
	ii. Sending children with symptoms to school	i. Susceptible to mental health illness;	ii. Unfamiliarity with healthcare system and services;
		iii. Unaware of mental health illness.	iii. Timing, channel and setting for healthcare services and information provision were important.
Service models*	A) A nurse with social work training will become the health/social care coordinator to provide health services information and call upon migrants to follow-up on their health seeking experience every 6 months.		
	B) Volunteers who are early migrants who have lived in Hong Kong for some time to be trained and form a support group to pay home visits to the migrants or providing them with health information.		D) Health workers or nurses organising peer education programmes with regular meeting for migrants screening behaviours for 1 year;
		C) Mental health “first aid” training and skills building workshops by psychologists or mental health nurses to provide training in self-esteem, emotional management and empowerment to increase the awareness on mental health problems;	

*The spread of the service models corresponds to the problems potentially addressed.

### Problems identified amongst migrants

#### Health literacy & communication

Based on their experiences in China, migrants commonly held different views on health and their expectations of health services in Hong Kong also differed from that of Hong Kong healthcare providers. As an example, one family physician from the FGD described how some migrants did not understand the difference between primary and secondary care, and therefore often requested unnecessary specialist referrals.

*“It is related to their upbringing- they often visit specialists (in China) even for small problems. They would request for specialists care even though the problems could be treated in primary care” (Family Physician).*

One teacher in the FGD responded that migrant parents who lacked the concept of primary care might also engage more in doctor-shopping or choose to self-medicate their sick children. Another doctor contended he tried to educate his migrant patients on the concept of primary care and that relationship building was essential in the process. However, it was further agreed that it was particularly difficult to develop professional relationships when “doctor-shopping” was commonly observed in migrant patients.

*“Perhaps they (the migrant mothers) do not have the concept of family doctor, even do not know where to see doctors- where to take their kids for consultations. They might try different doctors because they did not know which doctors would be good for them. Therefore, they would visit the public hospital, private clinic or try self-medication” (Teacher).*

And;

*“Perhaps after the doctors have built relationship with them, you can talk to them gradually, you can explain more, with some struggles, sometimes you may even need to argue with them… for these groups, they need to see private doctors, doctor shopping is quite common amongst them” (Family Physician).*

The levels of language and health literacy were not raised as a concern with our sample population. It was observed that some of the PCAs or staff were migrants themselves and could help facilitate communication between migrants and healthcare professionals.

#### Mental health and social support

The participants explained in the FGD that there was paucity of opportunity for migrants to build social networks among neighbours or colleagues in Hong Kong. This lack of networking opportunity combined with acknowledged tense familial relationships was seen to result in stress, potentially leading to depression or adjustment disorders.

Furthermore, many migrants and their family members were not aware that they had mental health problems. Notably, a social worker from the FGD held the impression that most of the migrants were quite receptive to mental health service:

*“In fact migration has already brought tremendous stress on the migrants. I seldom see the migrants telling us about their physical problems, but quite a lot of psychological problems were reported. It seems that they do not worry about the related stigma, i.e. Hong Kong people are afraid of the stigma attached to psychological problems, but they do not. They will see psychiatric specialists… Some of them have told me that they were depressed, could not sleep, and could not eat” (Social Worker).*

### Accessibility and appropriateness of health service and information

Time, cost, financial factors and lack of familiarity with health services were found to have played significant roles in migrants’ access to healthcare. Living in outer suburban areas and having to travel long distances to access healthcare institutions were seen by the FGD as a barrier to accessing care among migrants. FGD members further alluded to have observed that most of the new migrants were preoccupied with daily chores and economic hardship, which in turn led to a diminished awareness of their own health issues. Therefore, attention to access, affordability and operational hours of health services, as well as the setting in which healthcare information is delivered were all considered imperative to the subsequent utilisation of such services.

### Findings from the Delphi study

#### Key characteristics of healthcare models

##### Professional advice and signposting

After two rounds of Delphi questionnaires, our panel members reduced the four prior proposed service models to two models, A & C (Table 3). Further recommended was that the recruitment of a degree-holding registered nurse with post graduate qualifications in social work training (Model A), would be the most effective way to deliver and improve migrants’ health literacy. The nurse could direct the migrants to the appropriate service and guide them through different options of health and social service options such as private, non-governmental organisations, Hospital Authority, or Chinese medicine clinics. The panel members also pointed out the importance of having the same person provide continuity of care which would allow targeted monitoring to support health information uptake, understanding and positive health practices of the migrant population.

**Table 3:** **Quality and characteristics of proposed service models in Delphi questionnaire**

**Qualities or characteristics specific to particular service model**	
**Service model A**	**Service model B**
i. Professional;	i. Native and personalised services;
ii. Follow up and continuous monitoring on the	ii. Snowball effect and spread of mouth;
iii. health seeking behaviours of the migrants;	iii. Lower cost, better sustainability;
iv. Comprehensive advice for migrants to specific professional advice;	iv. Better understanding and higher sensitivity to their needs will lead to better acceptance toward the service by the migrants.
v. Counselling service to address physical and psychosocial issues.	
**Service model C**	**Service model D**
i. Professional;	i. More coverage, higher frequency;
ii. Empowerment would have long term effect on mental health.	ii. Peer educators will have better understanding of migrants’ problem;
	i. Long term health promotion will result in cost effectiveness.

Another service model which involved professional advice was that of a mental health first aid training workshop for migrants (Model C). Responses from the Delphi questionnaire emphasised that bridging the gap between knowledge and practice in mental health service access for the migrant population was important. Empowering migrants to utilise services that enhance their mental well-being would be an essential component of Model C.

##### Empowerment

The expert panel stressed the importance of an empowerment model for migrant health in their replies to the Delphi questionnaire. Promoting a sense of empowerment could exert long-term effects on migrants’ mental health and well-being. An empowerment model was seen to enable higher levels of acceptance and trust of health services and could be facilitated by the volunteers who were also from Mainland China with similar cultural and linguistic backgrounds. Similarly the experts believed that utilising volunteers in service model B or peers in service model D would enhance understanding, empathy and a heightened sensitivity to the migrants’ needs because these volunteers might themselves have faced similar problems in the past.

The social bonds formed among the group members from an empowerment service provision approach could provide the foundation for building long-term and sustainable social networks. Such networks could enable the effective channelling of peer support and health knowledge among the migrant population, with the potential to target hard-to-reach migrant populations and conduct further research. Responses from the questionnaire highlighted the possibilities of combining Models A & B in order to maximise the benefits from professional health advice and information, as well as the long-term social support. Moreover, the alignment of a social worker role with a community nursing role was seen as a desirable outcome.

## Discussion

This study contributes to the limited evidence of healthcare service utilisation research on migrants in a number of ways. First, our results affirm, that poor health literacy among migrants can be manifested by a lack of the understanding of the local healthcare system whilst having different expectations and opinions towards care and treatment. The contention being that these factors can have significant impacts on how Hong Kong migrants utilise healthcare services [3,21,22]. Language barriers are perceived to play a lessor role according to our targeted subjects in Hong Kong compared to other reports in the literature [3,22]. In this survey the migrants were seen to have lacked knowledge and awareness of mental illnesses, yet were more susceptible to familial and societal stigma and discrimination. It is further contended that shame and disgrace resulting from discrimination would predispose them to additional distress and possibly lead to further mental disorders and associated problems [15,23,24].

For migrants, gender also plays a major role in shaping individual and, at a macro-level, population health seeking behaviour [25]. Clearly it is important to maximise migrants’ access to health services and engage available social capital in order to meet the supply and demand sides of health service utilisation [26]. The assumption being that maximising the utilisation of services can only be promoted if both migrant men and women have accessible, acceptable and equitable service provision. We argue that, to enable Chinese migrants to engage more fully with health services and health decision-making in Hong Kong, they need to perceive the associated benefits from a targeted formal healthcare system [25,26]. Also of note is that migrants, in particular female spouses new to Hong Kong, may lack the necessary knowledge and skills needed to navigate the complex, foreign and commonly stigmatising healthcare system. This is further compounded by migrants’ diminished social capital and lack of targeted social resources, which, in turn, serve to discourage them from accessing health services [3,5].

Evidence points to the importance of having a healthcare workforce who can understand the health and welfare needs of the specific but otherwise vulnerable migrant population. The provision of such a workforce would serve to bridge the identified barriers to health-seeking behaviours and establish trust in the health services [3,22]. The importance of incorporating administrative and allied social welfare support staff into the existing teams of health professionals would enable an increased uptake of health information within currently limited consultation times [22]. We contend that a community-based nursing service with social work role capacity should be established to serve Mainland Chinese migrants’ health and social welfare needs. Such a service could provide both therapeutic relationships and continuity of care. Further long-term research is required to demonstrate that a community nursing model would facilitate the flow of health education information and bridge the gap for migrants most in need. At the same time it is important to have sufficient capacity of these multi-disciplinary qualified professionals to provide the service.

Other possible approaches to the empowerment service model include equipping the core formal health service with an outreach team or partnership with the lay healthcare workers or NGOs. This approach has been proven to be cost-effective in various migrant service healthcare settings and associated target populations [27,28]. The synergy of social empowerment through targeted personal support and health information has been shown to be effective for improving health literacy and health status in the low socio-economic and disadvantaged groups. The empowerment component embedded in the delivery model maximises social networks and support which are particularly salient for the migrant population [29].

However, there are a few limitations in this study. Although there is no universally agreed upon criteria or number provided for the selection of experts when applying Delphi technique [30], the fact that there were only one migrant and one patient representative could have affected the richness and diversity of the results gathered and perhaps a larger group of migrants or patients would have drawn more perspectives for the studied topic. However, we prioritised representativeness through diversity and range of migrants’ service experience in our panel member composition. These experts were also encouraged to gather the opinions from their frontline colleagues and these were then triangulated by the investigative team when they conducted site visits to the selected hospitals and clinics. The Delphi Technique was only able to observe quasi-anonymity, *i.e.* the experts would not know who had responded; thus, no one could exert overarching influence on another’s responses to the questionnaire [31].

## Conclusion

Chinese migrants were viewed by the expert panel as having poor health and a low rate of local healthcare service when compared to the local population due to having differing health understanding and expectations of the healthcare system. Many migrants were seen to be unaware of their own health needs and lacked insight on where health care could be sought. An empowerment based service model, if combined with the utilisation of specifically trained community nurse and volunteer groups, is recommended to improve health literacy and establish continuity of care through a trust-based relationship.

### Ethics approval of research

This study was approved by the Institutional Review Board of the University of Hong Kong/ Hospital Authority (UW 12–103).
